# Prevalence and correlates of previous adult imprisonment among Australians who primarily smoke methamphetamine: a cross-sectional study

**DOI:** 10.1186/s12954-025-01307-8

**Published:** 2025-10-03

**Authors:** Anna Peters, Bernadette Ward, Rebecca Kippen, Michael James Leach, Michael Curtis, Paul Dietze

**Affiliations:** 1 School of Rural Health, Monash University, Bendigo, VIC Australia; 2 Burnet Institute, Melbourne, VIC Australia; 3 National Drug Research Institute, Curtin University, Melbourne, VIC Australia

**Keywords:** Meth/amphetamines, Imprisonment, Stimulants, Criminal justice, Social determinants

## Abstract

**Background:**

In Australia, methamphetamine use is a significant public health concern, and is common among people involved with the criminal justice system. This study aimed to investigate the prevalence and correlates of adult imprisonment history among adults who primarily smoke methamphetamine.

**Methods:**

A cross-sectional study was conducted using baseline data from ‘VMAX’, a cohort of adults who regularly use methamphetamine. Data were collected between June 2016 and March 2020 from 718 participants. Sampling methods included convenience and respondent-driven sampling. Prison exposure was measured by asking if participants had ever been imprisoned due to a conviction (and was distinguished from juvenile detention). Logistic regression was used to examine how this correlated with socio-demographics, drug use, mental health, and criminogenic characteristics.

**Results:**

Nearly one-third (30%) of 718 participants reported having been imprisoned. Increased odds of reporting a history of imprisonment were found for participants reporting older age, male gender, non-metropolitan residential location, past-year homelessness, not being currently employed, schooling *≤* Year 9, *≥* weekly methamphetamine use, past-year illicit opioid use, injecting drug use history, and juvenile detention history. In contrast, participants reporting past-year other illicit stimulant (cocaine, ecstasy, illicit pharmaceutical stimulant) use were less likely to report a history of imprisonment.

**Conclusions:**

Social characteristics, patterns of drug use, and juvenile detention history were found to be correlated with imprisonment history. These findings point to the importance of providing targeted services to address characteristics of social disadvantage and drug use behaviours among people who use drugs, including among people who primarily smoke methamphetamine.

**Supplementary Information:**

The online version contains supplementary material available at 10.1186/s12954-025-01307-8.

## Background

Methamphetamine use is a significant public health concern in Australia [1]. Crystal methamphetamine (‘ice’; often a more potent form sold as crystals/shards [2]) has replaced powder methamphetamine (‘speed’) as the most commonly available and used form of methamphetamine, and the purity-adjusted price of all forms of the drug declined markedly from 2009 [3]. In turn, the way people use methamphetamine has changed, with a shift away from injecting and towards smoking as a preferred route of methamphetamine administration [2]. Smoking methamphetamine is associated with some of the same risks as injecting methamphetamine, including dependence [2], poor mental and physical health, and criminal justice involvement [4]. Among people who inject methamphetamine, smoking appears to complement, rather than replace, injecting [5]. All of these factors have contributed to reports of increased methamphetamine-related harms in Australia [1].

Among surveyed people entering prison in Australia, methamphetamine is the most commonly reported drug used in the year before imprisonment [6]. There is a high estimated prevalence of recent methamphetamine use among people entering prison (43% indicating use in the previous 12 months) [6], compared to the general population (1.3% in the previous 12 months) [7]. There is also an increased risk of recidivism for people who use methamphetamine compared to their non-using counterparts [8]. However, little is known about the association between methamphetamine smoking route of administration (RoA) and incarceration.

Few studies have examined the health and social characteristics of people who smoke methamphetamine [9] and little is known about the prevalence and correlates of imprisonment in this group. Moreover, much of the current detailed Australian information about methamphetamine use has come from cross-sectional studies of people who use drugs in Australia’s capital cities [10, 11]. Nevertheless, household surveys and other data sources suggest an increase in the use of methamphetamine in non-metropolitan Australia, compared to metropolitan areas [12]. By including participants from both metropolitan and non-metropolitan parts of Victoria, this study addresses a gap in the research about methamphetamine use and possible associated harms in non-metropolitan areas of Australia.

Given the high prevalence of recidivism and reincarceration in Australia [6], and the poor health and social outcomes associated with imprisonment among people who use drugs [13], we aimed to identify the prevalence of, and factors associated with, previous adult imprisonment amongst a cohort of people who primarily smoke methamphetamine.

## Methods

### Study design and methods

Our cross-sectional study used baseline data from ‘VMAX’, a prospective study of over 800 people living in metropolitan or non-metropolitan Victoria who regularly use methamphetamine. A more detailed description of VMAX has been published elsewhere [9]. Briefly, eligible participants were: aged *≥* 18 years; living in Melbourne or one of three non-metropolitan locations; using methamphetamine at least monthly in the six months prior to interview; and primarily using via a non-injecting route of administration. This final criterion was relaxed during recruitment to enable the study to reach its target sample size, meaning that some people in the cohort reported primarily or only injecting the drug [9].

Participants were recruited for annual VMAX surveys via a mixture of targeted, convenience, and respondent-driven sampling (RDS). Surveys administered by trained researchers collected data on participants’ socio-demographics, drug use characteristics, mental health, and criminal justice system contact data. Data were collected from June 2016 to March 2020 on tablets/laptops using Research Electronic Data Capture (REDCap) software [14]. Participants were reimbursed AUD40 upon completion of each survey.

### Dependent variable

The dependent variable analysed in this correlation study was a lifetime history of adult imprisonment, which was assessed by asking, ‘Have you ever been in prison due to a conviction/sentence?’, with dichotomised responses ‘Yes’ or ‘No’. This was differentiated from a history of juvenile detention, which was asked about in a preceding equivalent question.

### Independent variables

Variables which may be associated with a history of imprisonment were chosen for analysis based on a review of the literature which described factors associated with drug use and/or imprisonment. These were classed as demographic, social, methamphetamine use, other drug use, mental health, and criminal justice characteristics [15–19]. The variables analysed in this study, along with any validated tools used to measure them, are listed in Table 1.

**Table 1:** Independent variables, categories and tools used to measure them

Independent variable	Tool used	Categories
*Demographic characteristics*		
Age	N/A	18–29 years; 30–39 years; 40 + years. Also analysed as a continuous variable for descriptive statistics.
Gender	N/A	Male; Female.*
Aboriginal and/or Torres Strait Islander origin	N/A	Yes; No
Residential location	Modified Monash (MM) model [20]	Metropolitan (MM1); Non-metropolitan (MM2–MM5)
*Social characteristics*		
Past-year homelessness	N/A	Yes; No
Employment status	N/A	Currently employed**; Not currently employed
Highest schooling	N/A	≤ Year 9; ≥ Year 10
Social support	Enhancing recovery in coronary heart disease (ENRICHD) social support instrument [21, 22]	Low to medium (8–18); High (19–34)
*Methamphetamine use characteristics*		
Frequency of use	N/A	Less than weekly; Weekly or more
Preferred form	N/A	Crystal methamphetamine; Other^a^
Preferred route of administration	N/A	Smoking; Other^b^
Dependence	Severity of dependence scale (SDS) [23, 24]	Dependent (4–15); Not dependent (0–3)
*Other drug use characteristics*		
Alcohol use	Alcohol use disorder identification test – consumption (AUDIT-C) [25]	Abstinent (0); Moderate (1–7); High-risk (8–12)
Past-year other illicit stimulant^c^ use	N/A	Yes; No
Past-year cannabis use	N/A	Yes; No
Past-year illicit opioid^d^ use	N/A	Yes; No
Past-year illicit benzodiazepine use	N/A	Yes; No
History of injecting drug use	N/A	Yes; No
*Mental health characteristics*		
Anxiety	Generalised anxiety disorder-7 (GAD-7) [26]	None to mild (0–9); Moderate to severe (10–21)
Depression	Patient health questionnaire-9 (PHQ-9) [27]	None to mild (0–9); Moderate to severe (10–27)
*Criminal justice characteristics*		
Past-month property crime	N/A	Yes; No
Past-month drug selling	N/A	Yes; No
Past-month violent crime	N/A	Yes; No
History of juvenile detention	N/A	Yes; No

N/A = not applicable

* The small number of ‘other’ gender responses were set to missing, in line with other VMAX studies [9, 28, 29].

**Currently employed = Full-time, part-time, or casual.

^a^Other forms of methamphetamine: speed power, methamphetamine base, amphetamine liquid.

^b^Other routes of administration: injecting, snorting, swallowing, shelving/shafting.

^c^Other illicit stimulants: cocaine, ecstasy/methylenedioxymethamphetamine (MDMA), or illicit pharmaceutical stimulants.

^d^Illicit opioids: heroin, illicit pharmacotherapy (e.g. methadone, buprenorphine), or other illicit opioids (e.g. morphine, oxycodone).

### Statistical analysis

Data were analysed using a complete case approach, where participants with one or more missing variables were excluded from analysis [30]. Where the anxiety (GAD-7) and depression (PHQ-9) scales were partially answered, cases were included where the sum of answered items already exceeded 10 (i.e., already scored as having moderate to severe depression or anxiety) or where the maximum possible score of missing data could not change the category (i.e., seven of nine PHQ-9 items answered for a sum of two). Chi-square tests [31] and Mann-Whitney U tests [32] were used to determine if there were significant differences in characteristics between participants who were included and excluded on the basis of missing data.

Following descriptive analysis of the data, correlations between each independent variable and adult imprisonment were analysed using bivariable logistic regression, with results reported as odds ratios (OR) and 95% confidence intervals (CI). Statistical significance was set at *p* < 0.05. A multivariable model containing any fixed variables or variables that preceded adult imprisonment (age, gender, Aboriginal and/or Torres Strait Islander origin, juvenile detention, and education) was specified a priori and produced. Separate models comprising the a priori model and any one independent variable associated with incarceration at the 0.05 level in the preceding bivariable analyses were subsequently produced. Corrections for multiple comparisons were not performed due to the exploratory nature of the study.

All statistical analyses were performed using R version 4.1.2 (R Foundation for Statistical Computing, Vienna, Austria).

## Results

Eight hundred and forty-nine participants were recruited into VMAX; however, 131 participants (15%) were excluded from analysis due to missing data (2 due to missing lifetime adult imprisonment data and 129 due to missing covariate data), resulting in a final sample of 718 participants. Excluded participants were significantly more likely to identify as Aboriginal and/or Torres Strait Islander and have completed schooling to a Year 9 level or below (see Supplemental Table A).

Sample characteristics are shown in Table 2. The median age of the final sample was 34 (interquartile range 27–41) years; 60% identified as male, and 67% lived in a non-metropolitan location. Most (86%) participants used methamphetamine at least weekly. Crystal methamphetamine was the preferred form of methamphetamine for 94% of the sample, and 79% reported that smoking was their preferred route of administration. Nearly one-third of the sample (30%) reported a lifetime history of adult imprisonment. Of those respondents with a history of juvenile detention, 79% also reported a history of adult imprisonment.

**Table 2:** Sample characteristics (*N* = 718)

Independent variable	Categories	*n* (%)	History of adult imprisonment (% of each variable)
*Demographic characteristics*			
Age (Median = 34 years)	18–29 years	241 (33.6)	13.3
	30–39 years	261 (36.4)	30.7
	40 + years	216 (30.1)	48.6
Gender	Female	289 (40.3)	15.6
	Male	429 (59.7)	40.1
Aboriginal and/or Torres Strait Islander origin	No	620 (86.4)	27.9
	Yes	98 (13.6)	44.9
Residential location^a^	Metropolitan	237 (33.0)	13.1
	Non-metropolitan	481 (67.0)	38.7
*Social characteristics*			
Past-year homelessness	No	445 (62.0)	26.1
	Yes	273 (38.0)	37.0
Employment status	Currently employed	142 (19.8)	11.3
	Not currently employed	576 (80.2)	34.9
Highest schooling	≥ Year 10	523 (72.8)	23.3
	≤ Year 9	195 (27.2)	48.7
Social support^b^	Low to medium	228 (31.8)	30.3
	High	490 (68.2)	30.2
*Methamphetamine use characteristics*			
Frequency of use	Less than weekly	97 (13.5)	14.4
	Weekly or more	621 (86.5)	32.7
Preferred form	Other^i^	43 (6.0)	7.0
	Crystal methamphetamine	675 (94.0)	31.7
Preferred route of administration	Other^j^	152 (21.2)	36.2
	Smoking	566 (78.8)	28.6
Dependence^c^	Not dependent	243 (33.8)	31.4
	Dependent	475 (66.2)	28.0
*Other drug use characteristics*			
Alcohol use^d^	Abstinent	153 (21.3)	26.1
	Moderate	411 (57.2)	33.6
	High-risk	154 (21.4)	25.3
Past-year other illicit stimulant^e^ use	No	309 (43.0)	38.5
	Yes	409 (57.0)	24.0
Past-year cannabis use	No	143 (19.9)	23.1
	Yes	575 (80.1)	32.0
Past-year illicit opioid^f^ use	No	414 (57.7)	25.6
	Yes	304 (42.3)	36.5
Past-year illicit benzodiazepine use	No	384 (53.5)	27.6
	Yes	334 (46.5)	33.2
History of injecting drug use	No	316 (44.0)	13.6
	Yes	402 (56.0)	43.3
*Mental health characteristics*			
Anxiety^g^	None to mild	384 (53.5)	30.7
	Moderate to severe	334 (46.5)	29.6
Depression^h^	None to mild	346 (48.2)	30.3
	Moderate to severe	372 (51.8)	30.1
*Criminal justice characteristics*			
Past-month property crime	No	529 (73.7)	28.9
	Yes	189 (26.3)	33.9
Past-month drug selling	No	383 (53.3)	27.7
	Yes	335 (46.7)	33.1
Past-month violent crime	No	672 (93.6)	29.8
	Yes	46 (6.4)	37.0
History of juvenile detention	No	634 (88.3)	23.8
	Yes	84 (11.7)	78.6
History of adult imprisonment	No	501 (69.8)	–
	Yes	217 (30.2)	–

^a^Measured using Modified Monash Model.

^b^Measured using Enhancing Recovery in Coronary Heart Disease (ENRICHD) Social Support Instrument (ESSI).

^c^Measured using Severity of Dependence Scale (SDS).

^d^Measured using Alcohol Use Disorder Identification Test – Consumption (AUDIT-C).

^e^Illicit stimulants: cocaine, ecstasy/methylenedioxymethamphetamine (MDMA), illicit pharmaceutical stimulants.

^f^Illicit opioids: heroin, illicit pharmacotherapy, illicit prescription opioids.

^g^Measured using Generalised Anxiety Disorder-7 (GAD-7).

^h^Measured using Patient Health Questionnaire-9 (PHQ-9).

^i^Other forms of methamphetamine: speed powder, methamphetamine base, amphetamine liquid.

^j^Other routes of administration: injecting, swallowing, snorting, shelving/shafting.

The bivariable analyses, shown in Table 3, shows that the following factors were associated with increased odds of reporting previous adult imprisonment: older age, male gender, Aboriginal and/or Torres Strait Islander origin, non-metropolitan residential location, past-year homelessness, not being currently employed, year 9 or lower education, using methamphetamine at least weekly, crystal methamphetamine as preferred methamphetamine form, past-year cannabis use, past-year illicit opioid use, history of injecting drug use, and juvenile detention history. In contrast, participants reporting past-year other illicit stimulant (i.e. cocaine, ecstasy, illicit pharmaceutical stimulant) use were less likely to report a history of adult imprisonment.

**Table 3:** Results of bivariable logistic regression models: history of adult imprisonment (*N* = 718)

Independent variable	Categories	Bivariable models
*Demographic characteristics*		OR (95% CI)
Age	18–29 years	1.00
	30–39 years	2.89 (1.85, 4.60)*
	40 + years	6.18 (3.95, 9.89)*
Gender	Female	1.00
	Male	3.57 (2.50, 5.26)*
Aboriginal and/or Torres Strait Islander origin	No	1.00
	Yes	2.11 (1.36, 3.25)*
Residential location^a^	Metropolitan	1.00
	Non-metropolitan	4.19 (2.79, 6.47)*
*Social characteristics*		
Past-year homelessness	No	1.00
	Yes	1.67 (1.20, 2.30)*
Employment status	Currently employed	1.00
	Not currently employed	4.20 (2.50, 7.69)*
Highest schooling	≥ Year 10	1.00
	≤ Year 9	3.13 (2.22, 4.35)*
Social support^b^	Low to medium	1.00
	High	1.00 (0.71, 1.41)
*Methamphetamine use characteristics*		
Frequency of use	Less than weekly	1.00
	Weekly or more	2.88 (1.65, 5.41)*
Preferred form	Other^c^	1.00
	Crystal methamphetamine	6.19 (2.22, 25.78)*
Dependence^d^	Not dependent	1.00
	Dependent	1.18 (0.84, 1.66)
*Other drug use characteristics*		
Alcohol use^e^	Abstinent	1.00
	Moderate	1.43 (0.95, 2.18)
	High-risk	0.96 (0.57, 1.60)
Past-year other illicit stimulant^f^ use	No	1.00
	Yes	0.50 (0.36, 0.69)*
Past-year cannabis use	No	1.00
	Yes	1.57 (1.03, 2.43)*
Past-year illicit opioid^g^ use	No	1.00
	Yes	1.67 (1.21, 2.31)*
Past-year illicit benzodiazepine use	No	1.00
	Yes	1.31 (0.95, 1.80)
History of injecting drug use	No	1.00
	Yes	4.85 (3.35, 7.13)*
*Mental health characteristics*		
Anxiety^h^	None to mild	1.00
	Moderate to severe	0.95 (0.69, 1.31)
Depression^i^	None to mild	1.00
	Moderate to severe	0.99 (0.72, 1.36)
*Criminal justice characteristics*		
Past-month property crime	No	1.00
	Yes	1.26 (0.88, 1.79)
Past-month drug selling	No	1.00
	Yes	1.29 (0.94, 1.78)
Past-month violent crime	No	1.00
	Yes	1.38 (0.73, 2.55)
History of juvenile detention	No	1.00
	Yes	11.73 (6.89, 20.92)*

OR = odds ratio, CI = confidence interval, aOR = adjusted odds ratio

*p < 0.05

^a^ Measured using modified Monash model

^b^ Measured using enhancing recovery in coronary heart disease (ENRICHD) social support instrument (ESSI)

^c^ Other forms of methamphetamine: speed powder, methamphetamine base, amphetamine liquid

^d^ Measured using Severity of Dependence Scale (SDS)

^e^Measured using alcohol use disorder identification test – consumption (AUDIT-C)

^f^ Illicit stimulants: cocaine, ecstasy/methylenedioxymethamphetamine (MDMA), illicit pharmaceutical stimulants

^g^ Illicit opioids: heroin, illicit pharmacotherapy, illicit prescription opioids

^h^ Measured using generalised anxiety disorder-7 (GAD-7)

^i^ Measured using patient health questionnaire-9 (PHQ-9)

In the a priori model (Table 4), the following factors were associated with increased odds of reporting a history of adult imprisonment older age (age 30–39 years: aOR = 3.30, 95% CI = 2.00, 5.58; age *≥* 40 years: aOR = 5.52, 95% CI = 3.33, 9.37), male gender (aOR = 2.70, 95% CI = 1.82, 4.17), schooling *≤* Year 9 (aOR = 2.13, 95% CI = 1.43, 3.23), and juvenile detention history (aOR = 7.41, 95% CI = 4.07, 14.06).

When variables that were statistically significant in the bivariable analysis were analysed in multivariable analyses adjusting for the a priori model variables (Table 5), the following factors were associated with increased odds of reporting a history of adult imprisonment: living in a non-metropolitan area (aOR = 3.31, 95% CI = 2.05, 5.47), past-year homelessness (aOR = 1.72, 95% CI = 1.16, 2.53), not being currently employed (aOR = 3.13, 95% CI = 1.72, 5.88), at least weekly methamphetamine use (aOR = 3.07, 95% CI = 1.57, 6.49), past-year illicit opioid use (aOR = 1.48, 95% CI = 1.01, 2.15), and injecting drug use history (aOR = 2.76, 95% CI = 1.79, 4.32). Reporting past-year other illicit stimulant use (aOR = 0.61, 95% CI = 0.41, 0.89) remained associated with decreased odds of reporting a history of adult imprisonment in the multivariable model.

**Table 4:** Adjusted ORs of a priori base model: history of adult imprisonment (*N* = 718)

Independent variable	Categories	A priori model	
*Demographic characteristics*		aOR (95% CI)	
Age	18–29 years	1.00	
	30–39 years	3.30 (2.00, 5.58)*	
	40 + years	5.52 (3.33, 9.37)*	
Gender	Female	1.00	
	Male	2.70 (1.82, 4.17)*	
Aboriginal and/or Torres Strait Islander origin	No	1.00	
	Yes	1.68 (0.98, 2.87)	
*Social characteristics*			
Highest schooling	≥ Year 10	1.00	
	≤ Year 9	2.13 (1.43, 3.23)*	
*Criminal justice characteristics*			
History of juvenile detention	No	1.00	
	Yes	7.41 (4.07, 14.06)*	

^*^Denotes significance at *p* < 0.05

**Table 5:** Results of multivariable logistic regression models using an a priori base model: history of adult imprisonment (*N* = 718)

Independent variable	Categories	Multivariable analysis with adjustment from a priori model variables^a^
*Demographic characteristics*		aOR (95% CI)
Residential location^b^	Metropolitan	1.00
	Non-metropolitan	3.31 (2.05, 5.47)*
*Social characteristics*		
Past-year homelessness	No	1.00
	Yes	1.72 (1.16, 2.53)*
Employment status	Currently employed	1.00
	Not currently employed	3.13 (1.72, 5.88)*
*Methamphetamine use characteristics*		
Frequency of use	Less than weekly	1.00
	Weekly or more	3.07 (1.57, 6.49)*
Preferred form	Other^c^	1.00
	Crystal methamphetamine	2.92 (0.89, 14.25)
*Other drug use characteristics*		
Past-year other illicit stimulant^d^ use	No	1.00
	Yes	0.61 (0.41, 0.89)*
Past-year cannabis use	No	1.00
	Yes	0.99 (0.62, 1.60)
Past-year illicit opioid^e^ use	No	1.00
	Yes	1.48 (1.01, 2.15)*
History of injecting drug use	No	1.00
	Yes	2.76 (1.79, 4.32)*

^*^Denotes significance at *p* < 0.05.

^a^ Multivariable model adjusted for a priori model variables detailed in Table 4.

^b^ Measured using modified Monash Model.

^c^ Other forms of methamphetamine: speed powder, methamphetamine base, amphetamine liquid.

^d^ Illicit stimulants: cocaine, ecstasy/methylenedioxymethamphetamine (MDMA), illicit pharmaceutical stimulants.

^e^ Illicit opioids: heroin, illicit pharmacotherapy, illicit prescription opioids.

## Discussion

We found that 30% of a cohort of people who regularly use methamphetamine had a history of adult imprisonment. This finding is in accord with the high prevalence of methamphetamine use reported among those entering prison in Australia [6, 33] and is similar to a 2021 Australian study which found 28% of people who smoked and/or injected methamphetamine had a prison history [34]. However, among the participants in that study who mainly smoked methamphetamine, the prevalence was lower (22%). McKetin et al. also found that participants who mainly injected were more likely to have a prison history. Similarly, our study found a statistically significant correlation between injecting drug use history and imprisonment history, which is consistent with other studies showing injecting methamphetamine (either currently or previously) is associated with more risk for harms than other routes of administration [11, 34].

The prevalence of prison history found in our study is slightly lower than the prevalence of imprisonment (45%) found by McKetin et al. in a sample of people dependent on methamphetamine [5]. In contrast to our study participants, the McKetin et al. sample was comprised largely of people who injected the drug who were recruited before the dramatic market changes evident from 2009 [3]. In addition, their participants were recruited from treatment services, which may also be suggestive of more frequent or dependent methamphetamine use than in our community-recruited cohort. Moreover, their study did not differentiate between adult and juvenile detention history. These factors could all contribute to the higher reported prevalence of imprisonment history they found.

We found that living in a non-metropolitan part of Victoria, Australia was associated with increased odds of reporting a history of imprisonment. There is a well-established disparity in health, economic, and educational outcomes between people living in metropolitan and non-metropolitan areas of Australia [35]. These factors may contribute to the correlation between past imprisonment and non-metropolitan residential location in our sample. We found that the correlation persisted even when controlling for some characteristics of socioeconomic disadvantage (poor educational attainment, history of juvenile detention). There may be other factors mediating this correlation between imprisonment history and non-metropolitan residential location. Disadvantage experienced by people living in regional areas in Australia extends beyond socioeconomic factors, and includes poorer access to health services, intergenerational disadvantage, and higher prevalence of family violence and associated trauma [36]—factors that we did not measure but have since incorporated into our survey. There is a need for further exploration of factors that may contribute to imprisonment among people who use drugs in non-metropolitan areas, in order to better meet healthcare and social support needs for people who use methamphetamine outside of Australia’s capital cities.

Consistent with other studies among people who use drugs in Australia [16, 37], we found a correlation between imprisonment history and social disadvantage among people who use methamphetamine. Past-year homelessness, poor educational attainment, and not being currently employed were all significantly associated with increased odds of reporting adult imprisonment history. A high prevalence of characteristics of social disadvantage has been found among those in prison in Australia [6], and in other surveys of people who use methamphetamine [2, 5]. Social disadvantage, such as homelessness [38] and lack of employment [39–41], has been shown elsewhere to persist following imprisonment and is associated with harmful outcomes such as returning to prison [39] and/or returning to illicit drug use [40, 42, 43]. The correlation between social disadvantage and imprisonment history found in our study is not directional; further longitudinal research is required to ascertain whether social disadvantage is a predictor for, or consequence of, imprisonment.

Our study also found a correlation between juvenile detention history and adult imprisonment, highlighting the possibility of ongoing criminal justice involvement among people who use methamphetamine. This finding is consistent with high rates of reimprisonment and high rates of social disadvantage among people imprisoned as juveniles in Australia [15], demonstrating that this pattern is likely to persist among people using methamphetamine. These findings highlight the need for integrated health and social services for people who use methamphetamine, both preceding and following an episode of imprisonment.

Our study also found reports of past-year illicit opioid use associated with increased odds of imprisonment, but past-year illicit stimulant (excluding methamphetamine) use associated with decreased odds of reporting a history of imprisonment. This highlights how there is a relationship between polydrug use and harms such as imprisonment. In Australia, drug use is known to occur, and even be initiated, during imprisonment, with drugs such as heroin, illicit prescription drugs, and cannabis used more commonly than ecstasy [6, 44, 45]. Other Australian studies have shown there is a higher prevalence of imprisonment history among people who primarily use illicit opioids [11, 16] compared with people who regularly use ecstasy or other illicit stimulants [10]. Moreover, other characteristics of social disadvantage (such as unemployment, unstable housing, or poor educational attainment) are reported at much higher levels among Australian samples of people who inject drugs [11] compared to other samples such as people who regularly use ecstasy [10]. This disparity may contribute to differing criminal justice outcomes in these two groups. The correlation found in our study could indicate that certain patterns of drug use predispose to imprisonment among people who use methamphetamine; conversely, it could indicate that episode(s) of imprisonment lead to more intense polydrug use (i.e. illicit opioid use). Ongoing longitudinal work is needed to understand the intersection between the experience of drug-related harms and factors such as preferred route of methamphetamine administration, patterns of methamphetamine use, and the relationships with other illicit drug use.

### Strengths and limitations

A strength of this study is its relatively large sample size (*n* = 718), compared to other recent cross-sectional or cohort studies of people who use methamphetamine [5, 34]. Additionally, the inclusion of non-metropolitan participants in this study is a novel contribution to recent studies of methamphetamine use in Australia [5, 10, 11, 34].

Recruitment of the VMAX cohort is unlikely to have yielded a representative sample of people who use methamphetamine in Australia, particularly since non-metropolitan recruitment locations were selected based on evidence of methamphetamine-related harms in those areas. We had planned to incorporate tools for using RDS into the data analysis strategy to reduce recruitment biases but approximately half of the cohort were recruited using other methods, and we had incomplete network data for those who were recruited through RDS, so we were unable to determine the influence of sampling by comparing RDS and non-RDS samples. This will be addressed via ongoing data collection and analysis.

15% of our sample was excluded due to missing data. A disproportionate number of these missing data were from participants who identified as Aboriginal or Torres Strait Islander and/or who did not complete schooling past Year 9 (see Supplemental Table A). Our findings must be interpreted in light of this, potentially missing or underestimating correlations between these variables and imprisonment history.

Our study was limited by its cross-sectional design; we did not know whether imprisonment preceded methamphetamine (or other drug) use, or vice versa. Moreover, it is unclear from our study whether some of the independent variables preceded or followed an imprisonment episode. There is an opportunity for longitudinal research to ascertain the direction of the correlation between injecting drug use and imprisonment among people who use methamphetamine, in order to more effectively target prevention services.

Moreover, our question about imprisonment did not ascertain when, why or how many times participants were imprisoned. Additionally, the survey question only asked about convicted/sentenced episodes of imprisonment, so the prevalence of adult imprisonment history in this study is likely an underestimate. Participants may have been detained in a prison on remand without receiving a custodial sentence and thus were not captured by our survey question. Linkage with incarceration and arrest data is planned, with a view to building our understanding and treatment of people who use methamphetamine and are incarcerated.

Data on potential confounding factors (e.g. other mental illness [46, 47], cognitive dysfunction [47], or trauma [48]) were not collected in the survey but these have been shown to be associated with both methamphetamine use [49] and incarceration [50]. Further, longitudinal analyses are needed to examine the temporality of these associations. Moreover, there is the risk of recall and social desirability biases due to the sensitive nature of the questions.

## Conclusions

We found that 30% of a cohort of people who primarily smoke methamphetamine in Victoria, Australia reported a history of adult imprisonment. Adult imprisonment was associated with a range of factors linked to social disadvantage, drug use patterns, and previous criminal justice involvement. These findings demonstrate a need for increased social and health support services for people who use methamphetamine as a strategy to prevent a first episode of imprisonment, or to improve outcomes post-imprisonment.

## Supplementary Information

Supplementary Material 1

## Data Availability

The datasets used and/or analysed during the current study are available from the corresponding author on reasonable request.

## Abbreviations

aOR: Adjusted odds ratio
AUDIT-C: Alcohol use disorder identification test – consumption
CI: Confidence interval
ENRICHD: Enhancing recovery in coronary heart disease
GAD-7: Generalised anxiety disorder-7
MDMA: Methylenedioxymethamphetamine
MM: Modified Monash
OR: Odds ratio
PHQ-9: Patient health questionnaire-9
SDS: Severity of dependence scale

## References

[1] Man N, Sisson SA, McKetin R, Chrzanowska A, Bruno R, Dietze PM, et al. Trends in methamphetamine use, markets and harms in australia, 2003–2019. Drug Alcohol Rev. 2022;41(5):1041–52. 35604870 10.1111/dar.13468

[2] Degenhardt L, Sara G, McKetin R, Roxburgh A, Dobbins T, Farrell M, et al. Crystalline methamphetamine use and methamphetamine-related harms in Australia. Drug Alcohol Rev. 2017;36(2):160–70. 27286742 10.1111/dar.12426

[3] Scott N, Caulkins JP, Ritter A, Quinn C, Dietze P. High-frequency drug purity and price series as tools for explaining drug trends and harms in victoria, Australia. Addiction. 2015;110(1):120–8. 25220170 10.1111/add.12740

[4] Kinner SA, Degenhardt L. Crystal methamphetamine smoking among regular ecstasy users in australia: increases in use and associations with harm. Drug Alcohol Rev. 2008;27(3):292–300. 18368611 10.1080/09595230801919452

[5] McKetin R, Sutherland R, Peacock A, Farrell M, Degenhardt L. Patterns of smoking and injecting methamphetamine and their association with health and social outcomes. Drug Alcohol Rev. 2021;40(7):1256–65. 34365687 10.1111/dar.13364 PMC9292494

[6] Australian Institute of Health and Welfare. The health of Australia’s prisoners 2018, Summary [Internet]. Canberra (AU): AIHW; 2019 [cited 2023]. Available from: https://www.aihw.gov.au/reports/prisoners/health-australia-prisoners-2018/summary

[7] Australian Institute of Health and Welfare. National Drug Strategy Household Survey. 2019. Canberra (AU): AIHW, 2020 [cited 2023]. Available from: https://www.aihw.gov.au/reports/illicit-use-of-drugs/national-drug-strategy-household-survey-2019/contents/table-of-contents

[8] Cumming C, Kinner SA, McKetin R, Li I, Preen D. Methamphetamine use, health and criminal justice system outcomes: A systematic review. Drug Alcohol Rev. 2020;39(5):505–18. 32212214 10.1111/dar.13062

[9] Quinn B, Ward B, Agius PA, Jenkinson R, Hickman M, Sutton K, et al. A prospective cohort of people who use methamphetamine in Melbourne and non-metropolitan victoria, australia: baseline characteristics and correlates of methamphetamine dependence. Drug Alcohol Rev. 2021;40(7):1239–48. 33176047 10.1111/dar.13194

[10] Sutherland R, Uporova J, Karlsson A, Price O, Chandrasena U, Swanton R et al. Australian Drug Trends 2021: Key Findings from the National Ecstasy and related Drug Reporting System (EDRS) Interviews [Internet]. Sydney (AU); National Drug and Alcohol Research Centre, 2021 [cited 2023]. Available from: http://handle.unsw.edu.au/1959.4/unsworks_77989.

[11] Sutherland R, Karlsson A, King C, Jones F, Uporova J, Price O et al. Australian Drug Trends 2022: Key Findings from the National Illicit Drug Reporting System (IDRS) Interviews. [Internet]. Sydney (AU): National Drug and Alcohol Research Centre, 2022 [cited 2023]. Available from: http://handle.unsw.edu.au/1959.4/unsworks_81243.

[12] Roche A, McEntee A. Ice and the outback: patterns and prevalence of methamphetamine use in rural Australia. Aust J Rural Health. 2017;25(4):200–9. 27868256 10.1111/ajr.12331

[13] Csete J, Kamarulzaman A, Kazatchkine M, Altice F, Balicki M, Buxton J, et al. Public health and international drug policy. Lancet. 2016;387(10026):1427–80. 27021149 10.1016/S0140-6736(16)00619-X PMC5042332

[14] Harris PA, Taylor R, Thielke R, Payne J, Gonzalez N, Conde JG. Research electronic data capture (REDCap)--a metadata-driven methodology and workflow process for providing translational research informatics support. J Biomed Inf. 2009;42(2):377–81. 10.1016/j.jbi.2008.08.010 PMC2700030 18929686

[15] Indig D, Frewen A, Moore E. Predictors and correlates of re-incarceration among Australian young people in custody. Australian New Z J Criminol. 2016;49(1):73–89.

[16] Larney S, Cama E, Nelson E, Larance B, Degenhardt L. A cross-sectional study of correlates of imprisonment in opioid-dependent men and women in new South wales, Australia. Drug Alcohol Rev. 2016;35(6):686–92. 26711174 10.1111/dar.12357 PMC4927415

[17] Manning M, Wong GTW, Ransley J, Smith C. Analysing pseudoephedrine/methamphetamine policy options in Australia using multi-criteria decision modelling. Int J Drug Policy. 2016;32:85–92. 27179610 10.1016/j.drugpo.2016.03.012

[18] Sherman SG, Sutcliffe CG, Srirojn B, German D, Thomson N, Aramrattana A, et al. Predictors and consequences of incarceration among a sample of young Thai methamphetamine users. Drug Alcohol Rev. 2010;29(4):399–405. 20636656 10.1111/j.1465-3362.2009.00146.x

[19] Spooner C, Hetherington K. Social Determinants of Drug Use. Sydney (AU): National Drug and Alcohol Research Centre; 2004. Report No.: 228.

[20] Australian Government Department of Health and Aged Care. Modified Monash Model. Canberra (AU): Australian Government Department of Health and Aged Care, 2019 [cited 2023]. Available from: https://www.health.gov.au/topics/rural-health-workforce/classifications/mmm

[21] Burg MM, Barefoot J, Berkman L, Catellier DJ, Czajkowski S, Saab P, et al. Low perceived social support and post-myocardial infarction prognosis in the enhancing recovery in coronary heart disease clinical trial: the effects of treatment. Psychosom Med. 2005;67(6):879–88. 16314592 10.1097/01.psy.0000188480.61949.8c

[22] Vaglio J, Conard M, Poston WS, O’Keefe J, Haddock CK, House J, et al. Testing the performance of the ENRICHD social support instrument in cardiac patients. Health Qual Life Outcomes. 2004;2:24. 15142277 10.1186/1477-7525-2-24 PMC434528

[23] Gossop M, Darke S, Griffiths P, Hando J, Powis B, Hall W, et al. The severity of dependence scale (SDS): psychometric properties of the SDS in english and Australian samples of heroin, cocaine and amphetamine users. Addiction. 1995;90(5):607–14. 7795497 10.1046/j.1360-0443.1995.9056072.x

[24] Topp L, Mattick RP. Choosing a cut-off on the severity of dependence scale (SDS) for amphetamine users. Addiction. 1997;92(7):839–45. 9293043

[25] Bush K, Kivlahan DR, McDonell MB, Fihn SD, Bradley KA. The AUDIT alcohol consumption questions (AUDIT-C): an effective brief screening test for problem drinking. Ambulatory care quality improvement project (ACQUIP). Alcohol use disorders identification test. Arch Intern Med. 1998;158(16):1789–95. 9738608 10.1001/archinte.158.16.1789

[26] Spitzer RL, Kroenke K, Williams JBW, Löwe B. A brief measure for assessing generalized anxiety disorder: the GAD-7. Arch Intern Med. 2006;166(10):1092–7. 16717171 10.1001/archinte.166.10.1092

[27] Kroenke K, Spitzer RL, Williams JB. The PHQ-9: validity of a brief depression severity measure. J Gen Intern Med. 2001;16(9):606–13. 11556941 10.1046/j.1525-1497.2001.016009606.x PMC1495268

[28] Leach MJ, Ward B, Kippen R, Quinn B, Agius PA, Sutton K, et al. Level and correlates of social support in a community-based sample of Australians who primarily smoke methamphetamine. Health Soc Care Community. 2022;30(6):e4950–60. 35833453 10.1111/hsc.13907 PMC10946876

[29] Ward B, Kippen R, Reupert A, Maybery D, Agius PA, Quinn B, et al. Parent and child co-resident status among an Australian community-based sample of methamphetamine smokers. Drug Alcohol Rev. 2021;40(7):1275–80. 32896037 10.1111/dar.13155

[30] Altman DG, Bland JM. Missing data. BMJ. 2007;334(7590):424. 17322261 10.1136/bmj.38977.682025.2C PMC1804157

[31] Hazra A, Gogtay N. Biostatistics series module 4: comparing Groups – Categorical variables. Indian J Dermatol. 2016;61(4):385–92. 27512183 10.4103/0019-5154.185700 PMC4966396

[32] Hazra A, Gogtay N. Biostatistics series module 3: comparing groups: numerical variables. Indian J Dermatol. 2016;61(3):251–60. 27293244 10.4103/0019-5154.182416 PMC4885176

[33] Voce A, Sullivan T. Drug use monitoring in Australia: drug use among police detainees, 2020 [Internet]. Canberra (AU): Australian Institute of Criminology; 2021 [cited 2023]. Report No.: 35. Available from: 10.52922/sr78221

[34] McKetin R, Quinn B, Higgs P, Berk M, Dean OM, Turner A, et al. Clinical and demographic characteristics of people who smoke versus inject crystalline methamphetamine in australia: findings from a pharmacotherapy trial. Drug Alcohol Rev. 2021;40(7):1249–55. 33022140 10.1111/dar.13183

[35] Australian Institute of Health and Welfare. Rural and remote health [Internet]. Canberra (AU): Australian Institute of Health and Welfare; 2022 [cited 2023]. Available from: https://www.aihw.gov.au/reports/rural-remote-australians/rural-and-remote-health

[36] Tanton R, Dare L, Miranti R, Vidyattama Y, Yule A, McCabe M. Dropping Off the Edge 2021: Persistent and multilayered disadvantage in Australia [Internet]. Melbourne: Jesuit Social Services; 2021 [cited 2023]. Available from: https://www.dote.org.au

[37] Winter RJ, Stoové M, Agius PA, Hellard ME, Kinner SA. Injecting drug use is an independent risk factor for reincarceration after release from prison: A prospective cohort study. Drug Alcohol Rev. 2019;38(3):254–63. 30569550 10.1111/dar.12881

[38] Bashir AY, Moloney N, Elzain ME, Delaunois I, Sheikhi A, O’Donnell P, et al. From nowhere to nowhere. Homelessness and incarceration: a systematic review and meta-analysis. Int J Prison Health. 2021;17(4):452–61. 34107200 10.1108/IJPH-01-2021-0010

[39] Baldry E, McDonnell D, Maplestone P, Peeters M, Ex-Prisoners. Homelessness and the state in Australia. Australian New Z J Criminol. 2006;39(1):20–33.

[40] Winter RJ, Young JT, Stoové M, Agius PA, Hellard ME, Kinner SA. Resumption of injecting drug use following release from prison in Australia. Drug Alcohol Depend. 2016;168:104–11. 27635997 10.1016/j.drugalcdep.2016.08.640

[41] Stewart AC, Cossar RD, Wilkinson AL, Quinn B, Dietze P, Walker S, et al. The prison and transition health (PATH) cohort study: prevalence of health, social, and crime characteristics after release from prison for men reporting a history of injecting drug use in victoria, Australia. Drug Alcohol Depend. 2021;227:108970. 34488074 10.1016/j.drugalcdep.2021.108970

[42] Kinner SA. Continuity of health impairment and substance misuse among adult prisoners in queensland, Australia. Int J Prison Health. 2006;2(2):101–13.

[43] Curtis M, Winter RJ, Dietze P, Wilkinson AL, Cossar RD, Stewart AC, et al. High rates of resumption of injecting drug use following release from prison among men who injected drugs before imprisonment. Addiction. 2022;117(11):2887–98. 35665554 10.1111/add.15971 PMC9796148

[44] Kirwan A, Curtis M, Dietze P, Aitken C, Woods E, Walker S, et al. The prison and transition health (PATH) cohort study: study protocol and baseline characteristics of a cohort of men with a history of injecting drug use leaving prison in Australia. J Urban Health. 2019;96(3):400–10. 30989484 10.1007/s11524-019-00353-5 PMC6565648

[45] Butler T, Levy M, Dolan K, Kaldor J. Drug use and its correlates in an Australian prisoner population. Addict Res Theory. 2003;11(2):89–101.

[46] Degenhardt L, Coffey C, Hearps S, Kinner SA, Borschmann R, Moran P, et al. Associations between psychotic symptoms and substance use in young offenders. Drug Alcohol Rev. 2015;34(6):673–82. 26084677 10.1111/dar.12280

[47] Scott JC, Woods SP, Matt GE, Meyer RA, Heaton RK, Atkinson JH, et al. Neurocognitive effects of methamphetamine: a critical review and meta-analysis. Neuropsychol Rev. 2007;17(3):275–97. 17694436 10.1007/s11065-007-9031-0

[48] Bellis MA, Lowey H, Leckenby N, Hughes K, Harrison D. Adverse childhood experiences: retrospective study to determine their impact on adult health behaviours and health outcomes in a UK population. J Public Health (Oxf). 2014;36(1):81–91. 23587573 10.1093/pubmed/fdt038

[49] Duncan Z, Kippen R, Sutton K, Ward B, Rathnayake K, Quinn B, et al. Anxiety and depression among a community-recruited cohort of people who use methamphetamine: A longitudinal analysis. Addiction. 2025;120(4):697–710. 39545452 10.1111/add.16714

[50] Eaves ER, Camplain RL, Lininger MR, Trotter RT 2. nd. Adverse childhood experiences in relation to drug and alcohol use in the 30 days prior to incarceration in a County jail. Int J Prison Health. 2021;17(2):142–55. 34745314 10.1108/ijph-06-2020-0038 PMC8568314

