# Supplementary material for: Prevalence and correlates of previous adult imprisonment among Australians who primarily smoke methamphetamine: a cross-sectional study
Source: Harm Reduct J. 2025 Oct 3;22:157. doi: 10.1186/s12954-025-01307-8 (PMC12495676; doi:10.1186/s12954-025-01307-8)
Supplement: Supplementary file 1 — Supplementary Material 1 [file 12954_2025_1307_MOESM1_ESM.docx]

# Supplemental Table A

Summary statistics of VMAX variable of interest and covariates included in the multivariable logistic regression model, stratified by whether participants were included or excluded on the basis of missing data for other variables.

| **Characteristic** |  | **Included (n = 718),** n (%) | **Excluded (n = 131),** n (%) | ***p*-value** |
| --- | --- | --- | --- | --- |
| **Outcome** | | | | |
| Adult prison history | Yes | 217 (30%) | 42 (32%) | 0.67^a^ |
|  | No | 501 (70%) | 89 (68%) |  |
| **Covariates** | | | | |
| Age in years (median, IQR) |  | 34 (27, 41) | 34 (29, 42) | 0.23^b^ |
| Gender | Male | 429 (60%) | 74 (56%) | 0.49^a^ |
|  | Female | 289 (40%) | 57 (44%) |  |
| Aboriginal or Torres Strait Islander origin | Yes | 98 (14%) | 44 (34%) | < 0.01^a^* |
|  | No | 620 (86%) | 87 (66%) |  |
| Juvenile prison | Yes | 84 (12%) | 17 (13%) | 0.68^a^ |
|  | No | 634 (88%) | 114 (87%) |  |
| Schooling | < Year 9 | 195 (27%) | 51 (39%) | <0.01^a^* |
|  | > Year 10 | 523 (73%) | 80 (61%) |  |

^a^ Chi-square test

^b^ Mann-Whitney U Test

* Statistically significant at *p*<0.05 level

IQR = inter-quartile range
